# Accounting for gene flow from unsampled ghost populations while estimating evolutionary history

**DOI:** 10.1093/g3journal/jkaf180

**Published:** 2025-08-11

**Authors:** Arun Sethuraman, Melissa Lynch, Margaret Wanjiku, Michael Kuzminskiy

**Affiliations:** Department of Biology, San Diego State University, San Diego, California 92182, United States; Department of Biological Sciences, California State University San Marcos, San Marcos, CA 92096, United States; Department of Biology, San Diego State University, San Diego, California 92182, United States; Department of Biology, San Diego State University, San Diego, California 92182, United States

**Keywords:** Bias, Evolutionary History, Ghost Populations, Populations Genetics, Theory

## Abstract

Gene flow from unsampled or extinct ghost populations leave signatures on the genomes of individuals from extant, sampled populations, often introducing biases, data misinterpretation, and ambiguous results when estimating evolutionary history from population genomic data. Here we establish theoretical expectations for these biases, and then utilize extensive simulations under a variety of ghost topologies to systematically assess biases while accounting, or not accounting for gene flow from ghost populations in (i) population genetics summary statistics such as *π*, $F_{\mathrm{ST}}$, and Tajima’s *D* and (ii) demographic history (mutation-scaled effective population sizes, divergence times, and migration rates) under the Isolation with Migration (IM) model. Estimates of evolutionary history across all scenarios of deep divergence of an outgroup ghost indicate consistent (i) under-estimation of divergence times between sampled populations, (ii) over-estimation of effective population sizes of sampled populations, and (iii) under-estimation of migration rates between sampled populations, with increased gene flow from the unsampled ghost population. Without accounting for an unsampled ghost, summary statistics like $F_{\mathrm{ST}}$ are under-estimated, and *π* is over-estimated with increased gene flow from the ghost. These biases in summary statistics and population structure are however not captured under models of recent IM that approximate scales of the evolution of anatomically modern humans and Neanderthals and solely recapitulated using model-based estimation of evolutionary history. We also utilize a 355 locus dataset from African Hunter–Gatherer populations and discuss similar biases in estimating evolutionary history while not accounting for unsampled ghost.

## Teaser

Gene flow from unsampled ghost populations can bias estimates of population genetic summary statistics as well as model-based estimates of demographic history under the island and isolation with migration models. Here Sethuraman *et al*. (a) establish theoretical expectations, (b) utilize extensive simulations under models of differential gene flow from an unsampled ghost, and (c) utilize estimation of evolutionary history with a large empirical dataset from African Hunter-Gatherer populations to delineate these biases. The authors think that this will be an important contribution to the population and evolutionary genomics communities at large.

## Introduction

Population genomic data harbor signatures of evolutionary processes of drift, selection, and potentially gene flow between unsampled ghost and the sampled populations (Beerli 2004). Ghost populations comprise one or more extant or extinct populations that have, or continue to exchange genes with sampled populations. Such ghost populations are ubiquitous and have increasingly been discovered across numerous species complexes. For example, several studies of the evolutionary history of African hunter–gatherers (Lachance et al. 2012; Hey et al. 2018; Durvasula and Sankararaman 2020) identify significant gene flow from an unsampled archaic population (diverged from modern humans around the same time as Neanderthals) into multiple modern Hunter–Gatherer lineages. Similar studies in black rats (Konečnỳ et al. 2013), *Brachypodium sylvaticum* bunchgrass (Rosenthal et al. 2008), Blanding’s turtles (Sethuraman et al. 2014), bonobos (Kuhlwilm et al. 2019), and modern humans (Nielsen et al. 2017) also describe the presence of unsampled ghost populations that have exchanged genes with extant, sampled populations. Surprisingly, however, studies that account for unsampled ghosts are still scant in population genomics research and publications.

Not testing for the presence of, and thereon accounting for unsampled ghost population gene flow into extant sampled populations can lead to erroneous interpretation of population genetic summary statistics such as heterozygosity ($H_{e}$), population differentiation ($F_{\mathrm{ST}}$), nucleotide diversity (*π*), and Tajima’s *D*.

For instance, under a continent–island model, uncharacterized population structure involving an unsampled ghost will lead to overestimation of heterozygosity in sampled populations, often termed the Wahlund Effect. Similarly under an evolutionary scenario where two populations of a species are known to not directly exchange genes between each other but have exchanged genes bidirectionally with an unsampled ghost, measures of population differentiation ($F_{\mathrm{ST}}$) between sampled extant populations would be observed to be much lower than biologically expected under a null model (absence of a ghost).

Model-based methods for estimating population structure, unobserved allele frequency distributions, and gene flow, including Bayesian or maximum likelihood approaches, as implemented in software such as STRUCTURE (Pritchard et al. 2000), ADMIXTURE (Alexander et al. 2009), MIGRATE-n (Beerli and Felsenstein 2001), and IMa2p (Sethuraman and Hey 2016) are also not infallible to the effects of ghost gene flow, in that identical estimates of phylogenetic and mutational history may be achieved despite a number of unique demographic histories (Lawson et al. 2018). For example, Lawson et al. (2018) describe three scenarios of evolutionary history—one of recent admixture from four divergent populations, one of gene flow from a ghost population, and one of recent bottlenecks in sampled, extant populations. In the three different scenarios, the same population structure and admixture proportions are inferred using the programs STRUCTURE or ADMIXTURE. Correspondingly, not accounting for ghost gene flow has been known to bias estimates of migration rates (Hey et al. 2018) and divergence times (Lachance et al. 2012) while using other model-based estimators of evolutionary history. For instance, biases in estimates of effective population sizes and migration rates in the presence of the unsampled “ghost” populations were investigated under an Island Model (Beerli 2004; Slatkin 2005).

To quantify biases in the estimation of evolutionary history under more complex models, here we derive and establish theoretical expectations for biases under the Island model, and investigate the effects of varying degrees of ghost gene flow under the Isolation with Migration (IM) model (Nielsen and Wakeley 2001; Hey and Nielsen 2004, 2007). Both the Island and IM classes of models are widely used to model divergence with gene flow, where two or more sampled populations or subpopulations have diverged from an ancestor, and maintain gene flow via exchange of migrants postdivergence.

Additionally, as a proof of concept, we use 355 nuclear genomic loci under four demographic IM models to infer the demographic history of African Hunter–Gatherer populations to demonstrate these biases when not accounting for the presence of a ghost population. The evolutionary history of African Hunter–Gatherers has been previously summarized by Lachance et al. (2012), wherein a genome wide study of the Hadza and Sandawe indicated signals of (a) population bottlenecks in the Hadza, indicated by proportion of polymorphic sites and (b) ancient admixture determined by the S* statistic. Here, we utilize a model-based approach to understand these observations from the Hadza, Sandawe, Baka, and Yoruba to show how the addition of an unsampled ghost population aids in accurate estimates of demographic parameters using IMa3, comparable to the results of Hey et al. (2018).

## Theory

### Bias in estimates of genetic differentiation, measured as $F_{\mathrm{ST}}$

**Fig. 1.**
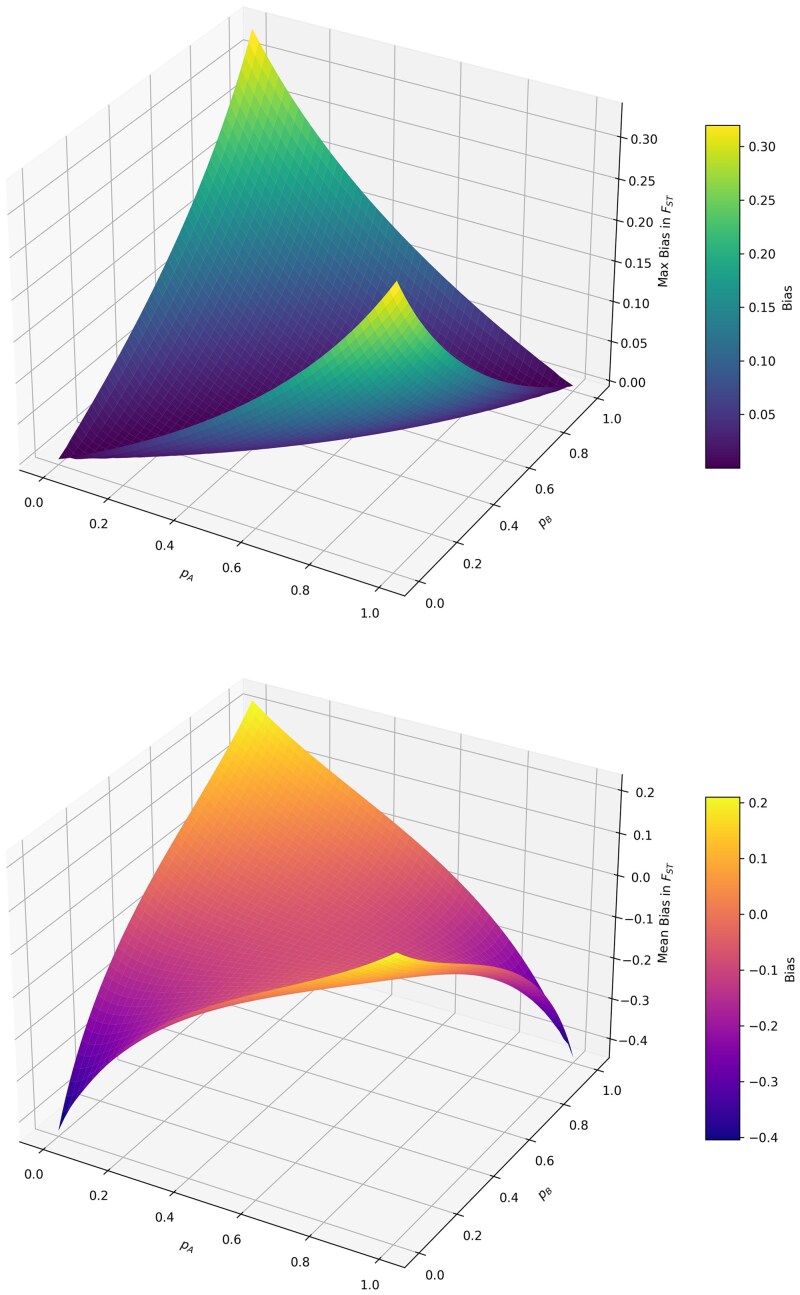
Theoretical (top) maximum bias distributions, and (bottom) mean bias distributions in estimates of $F_{\mathrm{ST}}$ under an island model without migration, computed across all values of allele frequencies in the ghost population $P_{C}$, and varying the allele frequencies in the sampled populations $P_{A}$ and $P_{B}$. Maximum and mean biases in estimates of $F_{\mathrm{ST}}$ are maximized when allele frequencies $P_{A},P_{C}$ are very different from each other, regardless of the allele frequency in the ghost population $P_{C}$.

Under an island model without migration, between population genetic differentiation can be estimated using the proportionate decrease in average expected heterozygosity while accounting for each subpopulation, compared to the expected heterozygosity in the total population (without accounting for subpopulation structure). Consider a case where there are three populations, A,B,C, where *A* and *B* are sampled populations and *C* is the third, unsampled ghost population. Let the frequency of an allele *A* at a diploid biallelic locus in these subpopulations be $p_{A},p_{B},\;\mathrm{and}\;p_{C}$ respectively. The average allele frequency, if computed using all three populations (truth) is then $\bar{\;p_{3}}=\frac{\;p_{A}+p_{B}+p_{C}}{3}$, while the same allele frequency computed using only the two sampled populations is $\bar{\;p_{2}}=\frac{\;p_{A}+p_{B}}{2}$. Therefore, the expected heterozygosity under Hardy–Weinberg equilibrium are computed as H(p)=2p×(1−p); meaning $H_{S}^{\left(2\right)}=\frac{1}{2}[2p_{A}(1-p_{A})+2p_{B}(1-p_{B})]$ using only the two sampled subpopulations, and $H_{S}^{\left(3\right)}=\frac{1}{3}[2p_{A}(1-p_{A})+2p_{B}(1-p_{B})+$  $2p_{C}(1-p_{C})]$. The expected heterozygosities of the total population, assuming no population structure then becomes $H_{T}^{\left(2\right)}=2\bar{\;p_{2}}(1-\bar{\;p_{2}})$ and $H_{T}^{\left(3\right)}=2\bar{\;p_{3}}(1-\bar{\;p_{3}})$. Now genomic differentiation can then be computed as $F_{\mathrm{ST}}^{\left(2\right)}=\frac{H_{T}^{\left(2\right)}-H_{S}^{\left(2\right)}}{H_{T}^{\left(2\right)}}$, and $F_{\mathrm{ST}}^{\left(3\right)}=\frac{H_{T}^{\left(3\right)}-H_{S}^{\left(3\right)}}{H_{T}^{\left(3\right)}}$. Therefore, bias in estimation of $F_{\mathrm{ST}}$ due to excluding the ghost population is $\mathrm{bias}=F_{\mathrm{ST}}^{\left(2\right)}-F_{\mathrm{ST}}^{\left(3\right)}$. We explore the bias distribution by applying boundary conditions to this scenario (Fig. 1), if $p_{A}\approx p_{B}$, bias is low, since there is no subpopulation structure, regardless of $p_{C}$. Bias also considerably increases when $p_{A}$ and $p_{B}$ are different from each other, and when the unsampled ghost population has $p_{C}$ in between $p_{A}$ and $p_{B}$.

**Fig. 2.**
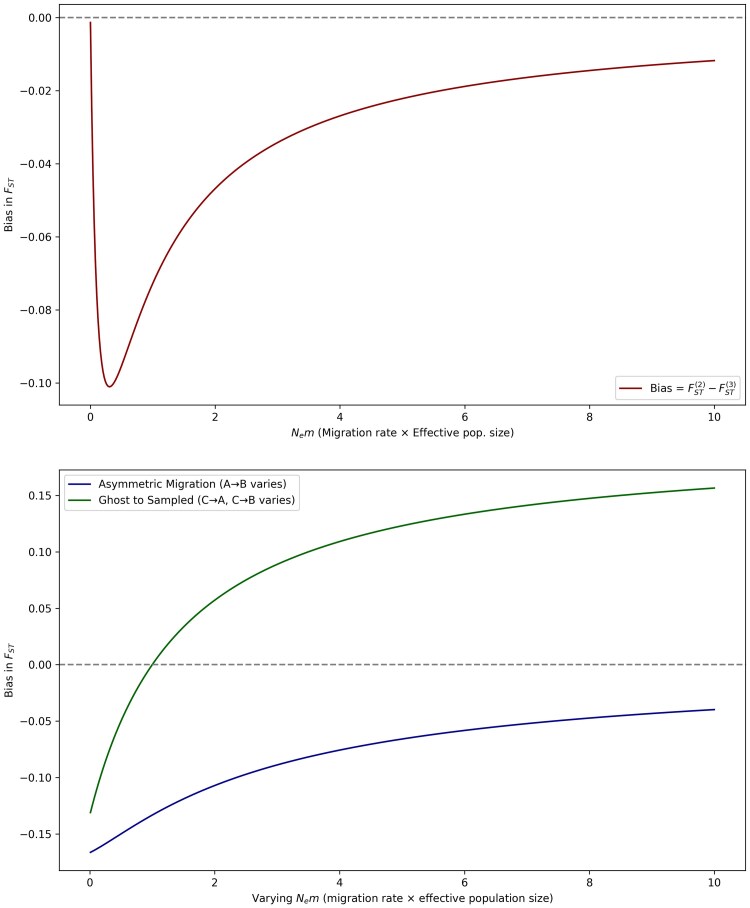
Theoretical bias distributions in estimates of $F_{\mathrm{ST}}$ (top) by varying $N_{e}m$, and (bottom) by varying the degree of asymmetric gene flow between the sampled populations A,B, and with the unsampled ghost population *C* under an island model with migration. Bias in estimated $F_{\mathrm{ST}}$ is minimized when $N_{e}m=0$, and increases negatively with (top) low $N_{e}m$, and diminishing again with larger $N_{e}m$ across all populations. Bias in $F_{\mathrm{ST}}$ however shows a different expected distribution under scenarios of asymmetric migration between the sampled populations A,B, and from the ghost population *C* (bottom).

Now if we add migration into the island model, such that *m* is the per generation migration rate, and $N_{e}$ is the effective population size of each population (A,B,C), $N_{e}m$ is the effective number of migrants per generation, and $F_{\mathrm{ST}}=\frac{1}{1+4N_{e}m}$ at drift-migration equilibrium. If we account for migration only between populations *A* and *B*, the per generation migration rate observable becomes $m_{\left(2\right)}=m$, while if we account for migration between all three populations, the per generation migration rate observable becomes $m_{\left(3\right)}=\frac{2}{3}m$, averaging the inflow and outflow of migration from two out of three possible sources. Therefore, $F_{\mathrm{ST}}^{\left(2\right)}=\frac{1}{1+4N_{e}m}$ while only accounting for the two sampled subpopulations, while $F_{\mathrm{ST}}^{\left(3\right)}=\frac{1}{1+\frac{8}{3}N_{e}m}$. Under this scenario, bias in estimation of $F_{\mathrm{ST}}$ due to excluding migration from the ghost population is $\mathrm{bias}=F_{\mathrm{ST}}^{\left(2\right)}-F_{\mathrm{ST}}^{\left(3\right)}$. Applying boundary conditions on *m*, we see that as m→0, $F_{\mathrm{ST}}^{\left(2\right)}\to 1$ and $F_{\mathrm{ST}}^{\left(3\right)}\to 1$, allowing bias=0. Similarly, m→∞, $F_{\mathrm{ST}}^{\left(2\right)}\to 0$ and $F_{\mathrm{ST}}^{\left(3\right)}\to 0$, allowing bias=0. Here, we simulate three scenarios (Fig. 2) to establish expectations and boundary conditions of the bias distribution of $F_{\mathrm{ST}}$: (a) variation of $N_{e}m$, here held constant across all three subpopulations, (b) variation in the asymmetric migration rate between sampled populations (A→B), and (c) variation in the migration rate between the sampled populations (A,B) and the ghost population (*C*), here measured as migration rates between C→A and C→B. Under scenario (a), we see that bias is maximized at intermediate values of $N_{e}m$ (moderate gene flow), while minimized as $N_{e}m\to 0$, and $N_{e}m\to \infty$. Under a scenario of asymmetric migration from A→B, we see that increased gene flow between the sampled populations reduces the bias in $F_{\mathrm{ST}}$, while increasing bias with addition of new alleles from the ghost population *C*. Under scenarios of gene flow from the ghost (C→A and C→B), we clearly see that bias in $F_{\mathrm{ST}}$ increases with increased gene flow from the ghost, showing how failing to model indirect gene flow from a shared migrant source (here the ghost, *C*) makes the sampled populations *A* and *B* look more different than they are.

### Bias in estimates of nucleotide diversity, measured as *π*

Under an island model without migration, here we use the definition of nucleotide diversity, measured as *π*, as the expected heterozygosity within a subpopulation, as explained in the previous section (i.e. $H_{S}^{\left(2\right)}$ and $H_{S}^{\left(3\right)}$), across a locus with *k* polymorphic sites; this equates to the average number of nucleotide differences per site between two randomly chosen sequences from a population; $\hat{\pi }=\sum_{i=1}^{k}2p_{i}(1-p_{i})$, where $p_{i}$ is the allele frequency at site *i*. Using the same definitions of bias as before, we compute ${\hat{\pi }}^{\left(2\right)}=\frac{1}{2}(\pi_{A}+\pi_{B})$, and ${\hat{\pi }}^{\left(3\right)}=\frac{1}{3}(\pi_{A}+\pi_{B}+\pi_{C})$, with $\mathrm{bias}={\hat{\pi }}^{\left(2\right)}-{\hat{\pi }}^{\left(3\right)}$  $=\frac{1}{2}(\pi_{A}+\pi_{B})-\frac{1}{3}(\pi_{A}+\pi_{B}+\pi_{C})=\frac{1}{6}(\pi_{A}+\pi_{B}-2\pi_{C})$. Applying boundary conditions here, the degree of bias in estimates of nucleotide diversity therefore depends on the nucleotide diversity of the ghost ($\pi_{C}$); omitting a more diverse ghost therefore will lead to underestimates of true $\hat{\pi }$.

**Fig. 3.**
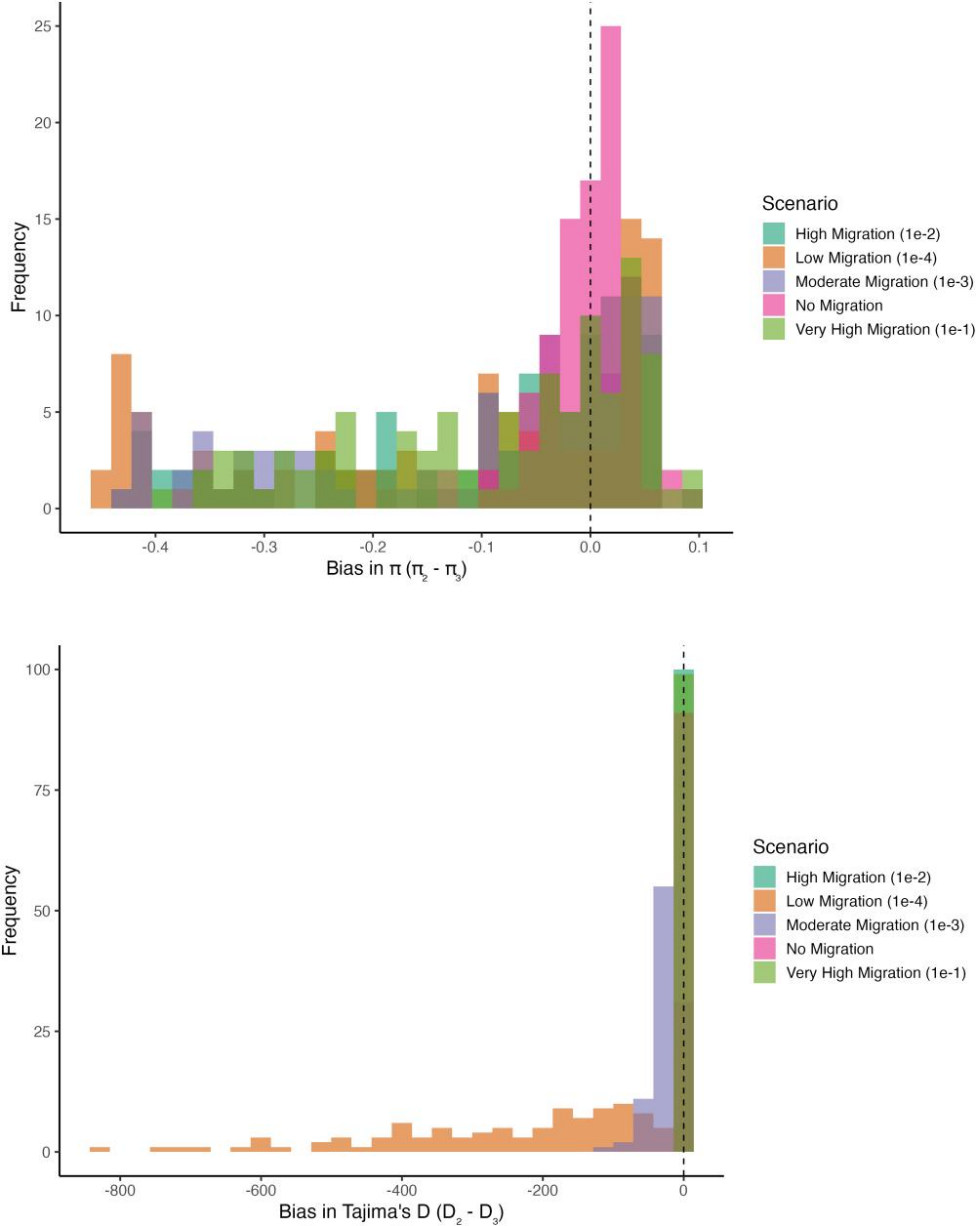
Theoretical bias distributions in (top) estimates of nucleotide diversity *π*, and (bottom) in estimates of Tajima’s *D* by varying the degree of asymmetric gene flow (*m*) between the sampled populations A,B, and with the unsampled ghost population *C*. Bias in estimates of *π* (top) are minimized when there is no migration (m=0), while showing increased variation and negative bias with increasing *m*. Bias in estimates of Tajima’s *D* are also minimized at m=0, negatively biased with low degrees of migration (m=0.0001), but the bias diminishes with higher migration (m≥0.001), returning to 0 at high migration rates (m=0.1).

Now if we add migration to this, wherein allele frequencies become more similar to each other with increased migration, and therefore increasing within population nucleotide diversity but decreasing between population differentiation, let’s define ${\bar{\pi }}_{\mathrm{within}}$ as the mean pairwise diversity within each subpopulation, and ${\bar{\pi }}_{\mathrm{total}}$ as the mean pairwise diversity across the total population, by pooling all individuals and including cross-subpopulation comparisons, then ${\bar{\pi }}_{\mathrm{pooled}}>{\bar{\pi }}_{\mathrm{within}}$ in the presence of subpopulation structure. If there is migration between subpopulations, ${\bar{\pi }}_{\mathrm{within}}$ increases due to alleles being shared between subpopulations, but ${\bar{\pi }}_{\mathrm{pooled}}$ can increase or decrease depending on the amount of population structure (i.e. differentiation between subpopulations). Similar to the above scenario without migration, then ${\hat{\pi }}^{\left(2\right)}=\frac{1}{2}(\pi_{A}+\pi_{B})$, and ${\hat{\pi }}^{\left(3\right)}=\frac{1}{9}\sum_{i,j\in \{A,B,C\}}\pi_{ij}$. Thereon, $\mathrm{bias}={\hat{\pi }}^{\left(2\right)}-{\hat{\pi }}^{\left(3\right)}=$  $\frac{1}{2}(\pi_{A}+\pi_{B})-\frac{1}{9}\sum_{i,j\in \{A,B,C\}}\pi_{ij}$. This bias will tend to be negative by ignoring the ghost, especially if we have a diverse and divergent ghost population. With increased migration from the ghost however, since the within population diversity increases, and between population diversity decreases, bias in nucleotide diversity estimation will also decrease over time. We simulate these two scenarios (no gene flow i.e. m=0, versus gene flow with the ghost population *C* i.e. m=0.0001−0.1—Fig. 3, top panel) to explore these boundary conditions. We see that the bias in *π* is more negative and more variable under m=0, indicating that the true nucleotide diversity is always underestimated. Under increased *m*, the bias is less negative and closer to 0, caused by allele frequencies to converge due to migration with the ghost.

### Bias in estimates of Tajima’s *D*, Watterson’s *θ*, number of segregating sites *S*

Here, we discuss the bias in estimates of Tajima’s *D*, which combines pairwise nucleotide diversity (discussed above) $\hat{\pi }$, and Watterson’s $\hat{\theta }$, which in itself is the number of segregating sites *S*, scaled by the number of pairwise comparisons. Tajima’s $D=\frac{\hat{\pi }-\hat{\theta }}{\sqrt{V}}$, where $\hat{\theta }=\frac{S}{\sum_{i=1}^{n-1}\frac{1}{i}}$, the denominator being the Stirling number function, and *V* is the variance (normalization term). With increased degree of subpopulation structure, $\hat{\pi }$ increases due to the accumulation of differences between subpopulations, while *S* increases slowly (mostly shared variation), making Tajima’s *D* more positive. If we ignore the presence of a ghost population, as discussed above, we underestimate $\hat{\pi }$ (due to fewer between-group differences) and *S* (due to just missing segregating sites from the ghost). Depending on the differences between these, we would therefore expect Tajima’s *D* to be more negative and biased downward.

We define bias as before—$\mathrm{bias}=D^{\left(2\right)}-D^{\left(3\right)}$, where $D^{\left(2\right)}$ is the Tajima’s D estimated with only the two sampled populations (A,B), and $D^{\left(3\right)}$ is the Tajima’s D estimated with all three populations, including the ghost (*C*). Here, we simulate the bias distribution under no migration, and a model of migration (Fig. 3, bottom panel); as expected, Tajima’s *D* estimates are biased negatively when not accounting for a ghost population, with increased gene flow from a ghost population dampening this bias.

## Methods

### Simulations

To address how unsampled ghost variation influences sampled genomes, we simulated genomic data under two scenarios: recent divergence (along the time scales of recent divergence of anatomically modern humans and neanderthals), and deep divergence (similar to the divergence timescales of anatomically modern humans and chimpanzees). All simulations were performed using *msprime* v.1.0 (Kelleher and Lohse 2020; Baumdicker et al. 2022), a coalescent simulator of demographic history and DNA sequence evolution based on *tskit* v.1.0 (Kelleher et al. 2018).

**Fig. 4.**
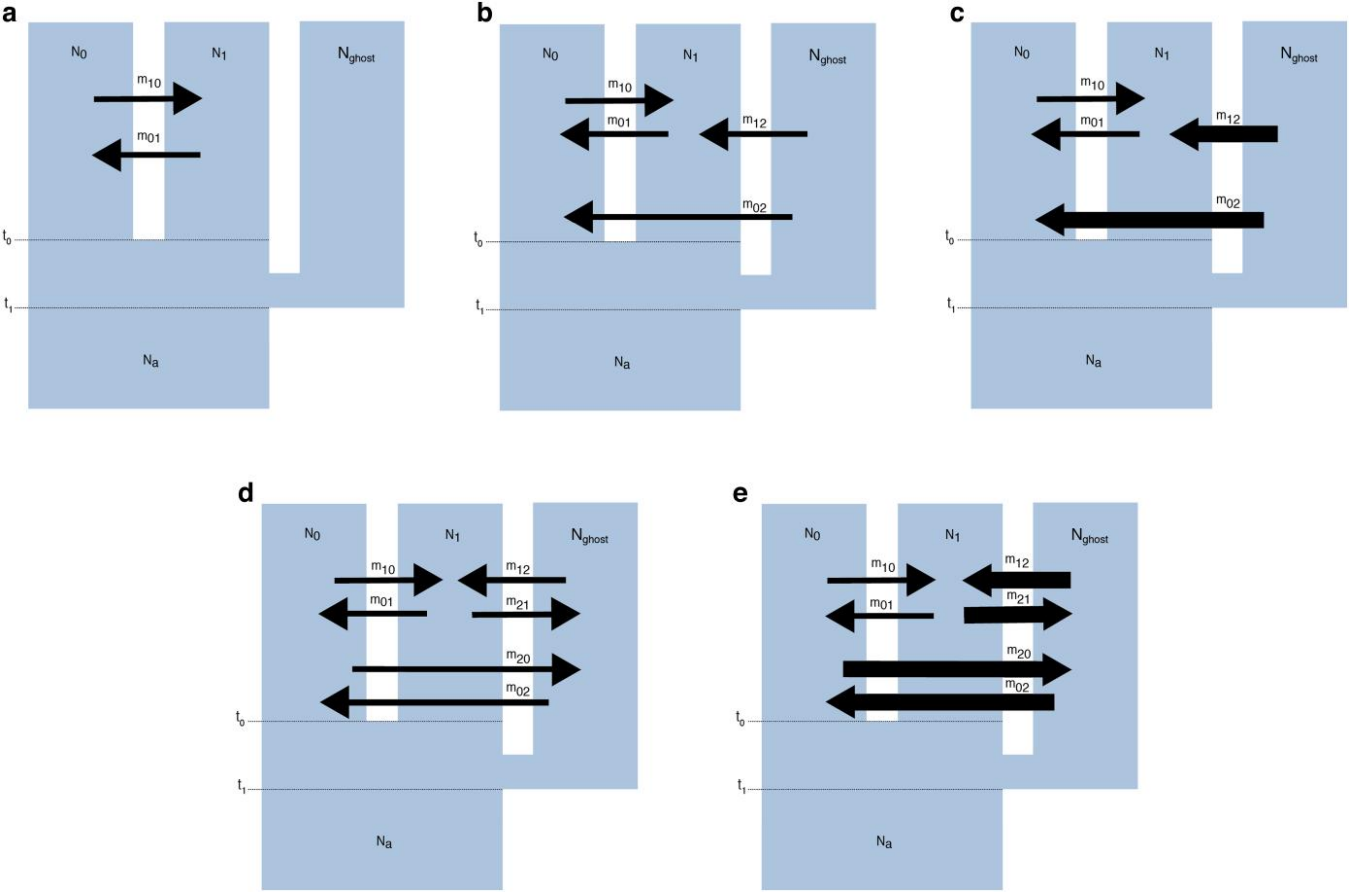
Models a–e under the Isolation with Migration model. $N_{0}$ and $N_{1}$ are effective population sizes of the two sampled populations (which diverged at time $t_{0}$), $N_{\mathrm{ghost}}$ is the effective population size of the unsampled ghost population, and $N_{a}$ is effective population size of the common ancestor of ${\text{N}}_{0}$, $N_{1}$ and ghost (which diverged at time $t_{1}$). The arrows indicate either high (bold arrow) or low (small arrow) levels of gene flow, as well as direction. Model a is the null model, under which there is no gene flow from the ghost into the two sampled populations, while models b and c represent low and high unidirectional gene flow from the ghost into the two sampled populations, respectively. Models d and e represent models of low and high bidirectional gene flow to and from the ghost into the two sampled populations, respectively. All models were simulated under both recent and deep divergence scenarios.

Data were simulated under 5 IM models (Fig. 4). Under model a, only populations $N_{0}$ and $N_{1}$ exchange genes since divergence from their common ancestor at a rate of $4N_{0}m_{0\to 1}=4N_{1}m_{1\to 0}$. Neither $N_{0}$ nor $N_{1}$ exchange any migrants with the ghost population $N_{\mathrm{ghost}}$. Under model b, $N_{0}$ and $N_{1}$ exchange migrants at a rate of 4$N_{0}m_{0\to 1}=4N_{1}m_{1\to 0}$ as in model a, but individuals also emigrate out of the ghost $N_{ghost}$ into $N_{0}$ and $N_{1}$ at the same rate (4$N_{\mathrm{ghost}}m_{1\to 2}=4N_{ghost}m_{0\to 2}$). Under model c, the ghost population $N_{\mathrm{ghost}}$ exchanges 4$N_{\mathrm{ghost}}m_{1\to 2}=4N_{\mathrm{ghost}}m_{0\to 2}$, while $N_{0}$ and $N_{1}$ continue to exchange migrants at the same low rate (4$N_{0}m_{0\to 1}=4N_{1}m_{1\to 0}$). Under model d, all three populations exchange migrants at the same low rate (4$N_{e}m$). Under the last model e, the ghost $N_{\mathrm{ghost}}$ exchanges migrants bidirectionally at a high rate (4$N_{e}m$), while the two sampled populations $N_{0}$ and $N_{1}$ still exchange migrants at a low rate of 4$N_{0}m_{0\to 1}=4N_{1}m_{1\to 0}$. All migration rates were specified backwards in time in coalescent simulations, and represent “where” the lineages coalesce; therefore the parameter estimates correspond to individuals migrating in the opposite direction, forwards in time (see https://tskit.dev/msprime/docs/stable/demography.html#sec-demography-migration for a detailed explanation of direction of migration during coalescent simulations).

Under all simulated models, 10 individuals were sampled from each population, and separate datasets were constructed with 20 unlinked genomic loci of length 1,000 bp. Ten replicate datasets were simulated under each model to construct confidence intervals around estimates. Low migration rates were simulated at m=0.05, while high migration rates were simulated at m=0.5. Recent divergence models began with an initial population ancestor with effective population size 5,000. At 1,850 generations (scaled by a modern human generation time of 29 years), this ancestor splits into the ghost population (ghost) and the common ancestor of the sampled populations. At1,850 generations, this common ancestor splits into populations 0 and 1. All populations were kept at an effective population size of 5,000. Comparable deep divergence models had an initial population split 68,966 generations ago, and a recent population split 17,241 generations ago (all scaled by the modern human generation time of 29 years). All other parameters were maintained across deep and recent divergence models. Mutations within individuals were then simulated at $1\times 10^{-8}$ mutations per generation per site, which is consistent with the human mutation rate per site per generation.

### Summary statistics

PPP v.1.0 (Webb et al. 2021) scripts were then utilized to estimate genomic differentiation (estimated as Weir and Cockerham’s $F_{\mathrm{ST}}$), nucleotide diversity (*π*), and Tajima’s *D* (to measure differences in the observed site frequency spectra due to rare allelic variation) under both recent and deep divergence scenarios. All code for simulations and computing summary statistics is available as msprime3pop.py in the project’s GitHub page.

### Estimates of evolutionary history—IM model

Evolutionary history under the deep divergence model was then estimated with IMa3 (Hey et al. 2018) in three separate ways: (i) a two population model, wherein the simulated genomic data was down-sampled to only include populations $N_{0}$ and $N_{1}$, (ii) a three population model, wherein all three populations, $N_{0}$, $N_{1}$, and $N_{\mathrm{ghost}}$ were included in a population model with $N_{0}$ and $N_{1}$ sharing a more recent common ancestor, and $N_{\mathrm{ghost}}$ as the outgroup and (iii) a two population model (same as model i), but with the addition of an outgroup ghost population. Model (iii) is the ideal scenario, wherein users can utilize the option of adding an unsampled ghost population to their estimation of evolutionary history. This option has been conveniently written into IMa2p (Sethuraman and Hey 2016) and IMa3 (Hey et al. 2018).

Prior values for effective population sizes, divergence times, and migration rates were set using estimates of Watterson’s *θ*. Upper bounds on *θ* estimates were set to be five times the geometric mean of Watterson’s *θ* across all loci, the upper bound on the divergence times were set to two times the geometric mean of Watterson’s *θ* across all loci, and the upper bound on migration rates was set to 2 divided by the geometric mean of Watterson’s *θ* across all loci, according to the recommendations of Hey (2011a).

All runs were performed using 10 chains distributed across 56 processors (total of 560 chains), discarding $1\times 10^{6}$ MCMC iterations as burn-in, followed by sampling 100,000 genealogies. It was ensured that all chains were sufficiently mixed by swapping genealogies across chains (swap rates >0.5) and that the chains had converged prior to sampling genealogies (inferred by observing the autocorrelations between parameter estimates, across iterations, and in effective sample size values).

All sampled genealogies were then used to estimate marginal posterior densities of evolutionary demographic parameters, and the modes of these marginal distributions and 95% confidence intervals around the modes were computed, and compared to the “true” simulated parameters used in msprime. IM analyses were also repeated by down-sampling the number of loci (2 versus 5) to assess robustness of inference with decrease in genomic data used for inference. Recent divergence models with gene flow were not analyzed under the IM model owing to known issues with identifiability of demographic parameters under recent divergence with gene flow (Cruickshank and Hahn 2014; Hey et al. 2015).

### Analyses of empirical data under the IM model

African Hunter–Gatherer populations (Hadza, Sandawe) are a culturally diverse group of indigenous populations who are hypothesized to have diverged from other ancient African modern human lineages (particularly the Pygmy (Baka) and Yoruba (agricultural) populations). The Hadza and Sandawe are both historically Hunter–Gatherer populations from Central Tanzania and are known to share a complex cultural and demographic history. While the Hadza are hypothesized to have little cultural confluence with other native populations, the Sandawe, on the other hand, have historically admixed with other Northern populations, including the Baka (pygmy), and Yoruba (pastoral/agricultural). Lachance et al. (2012) used summary statistics to determine signals of (i) population bottleneck in the Hadza (using the relative proportion of polymorphic sites) and (ii) archaic admixture with an unknown ancestral species or population, using the S* statistic.

We filtered the genomes of 20 individuals (5 each of Hadza, Sandawe, Baka, and Yoruba) with filters used by Gronau et al. (2011), based on removing recombination hotspots, duplications, syntenic regions with *P. troglodytes*. The filtered diploid SNP loci were then phased with fastPHASE to obtain haplotypes across populations Scheet and Stephens (2006). The haplotypes were then filtered further to remove possible recombining segments using a four-gamete test (Hudson and Kaplan 1985). This produced a total of 355 random, unlinked, putatively neutral loci, which were used in IM and admixture analyses.

Demography was then inferred under different models of population history—(i) 2 population IM models with all pairs of populations (Hadza–Sandawe, Sandawe–Baka, Baka–Yoruba, Baka–Hadza, Sandawe–Yoruba, Hadza–Yoruba) assuming that there is no ghost population (Fig. 9a) and (ii) 2 population IM models with all pairs of populations, assuming that there is an outgroup ghost population (Fig. 9b).

The ghost population is assumed to be an unsampled outgroup lineage that exchanges genes with sampled populations at constant migration rates, estimated under the IM model. All priors on population sizes, divergence times, and migration rates were set based on the guidelines of Hey (2011b), by using the harmonic mean of Watterson’s estimator of $N_{e}$ across loci (see Supplementary Table 6). Parallel runs of MCMC were then performed with 100,000 iterations of burn-in, followed by a total run time of 48 h. Mixing, and convergence were assessed by observing swap rates over runs, acceptance rates of parameter updates, effective sample sizes, and autocorrelations. Convergence of the MCMC was then assessed using Tracer (Rambaut et al. 2018). Sampled genealogies were used to estimate marginal posterior density distributions of demographic parameters and scaled with the modern human generation time of 29 years. Likelihood ratio tests were then used to determine statistical significance of migration estimates. These estimates were then compared with the four-population models (with ghost) of Hey et al. (2018), to understand potential parameter biases and ascertain power to provide support for or against the presence of unsampled ghost populations.

## Results

### Effects on the estimation of summary statistics

**Fig. 5.**
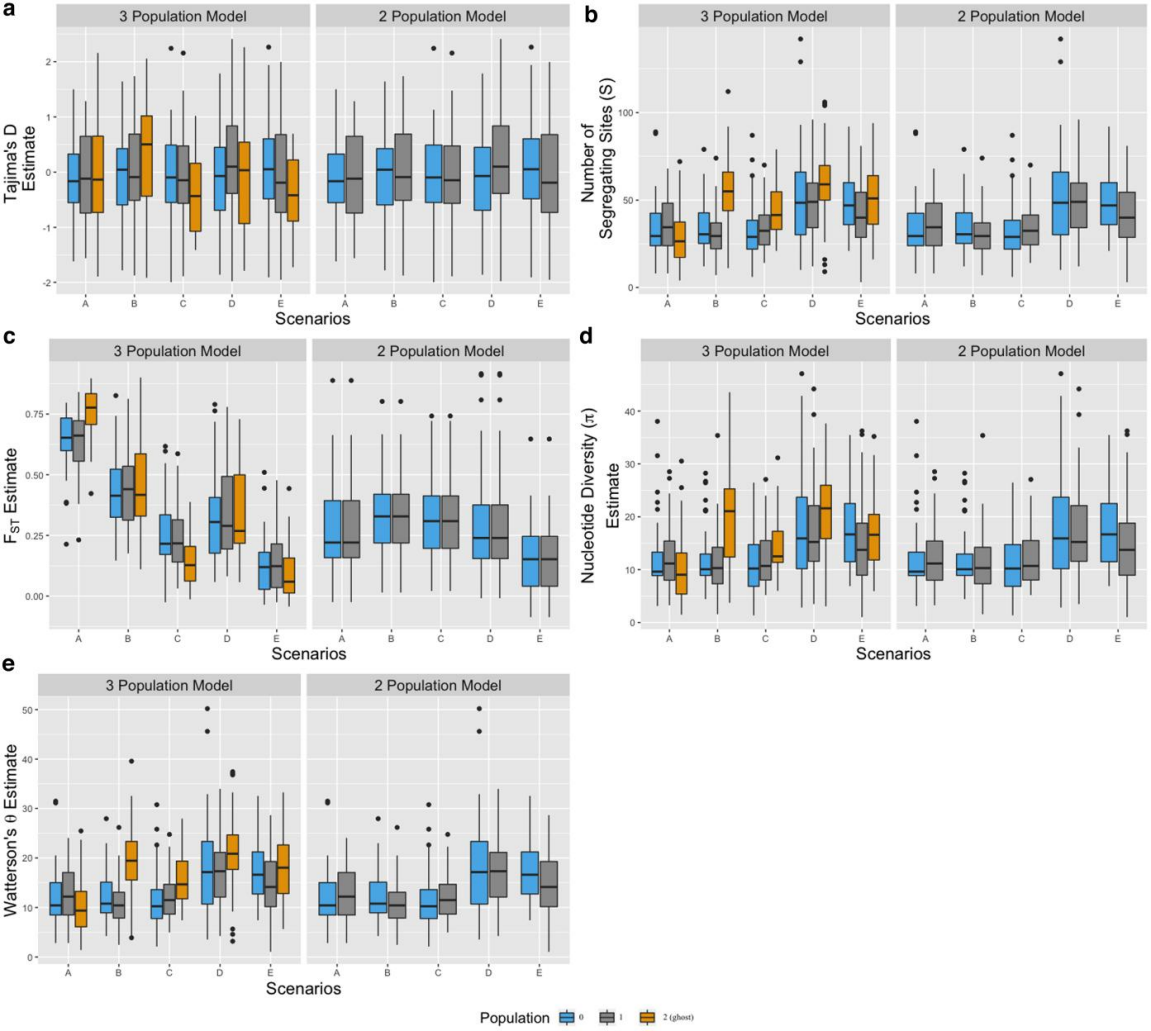
Summary statistic estimates under models a–e while. Under each model, we estimate a) Tajima’s *D*, b) number of segregating sites (*S*), c) Genetic Differentiation ($F_{\mathrm{ST}}$, d) nucleotide diversity (*π*), and e) effective population size (Watterson’s *θ*) under either a 3 population sampling strategy (left panels), or a 2 population sampling strategy (right panels). We would expect that all estimates of Tajima’s D would be close to 0 (indicating putatively neutral evolution), estimates of differentiation between the two sampled populations to be smaller with greater gene flow from the ghost (i.e. under models d, e, compared to model a), and estimates of nucleotide diversity to be greater with greater gene flow from the ghost. This is reflected in all estimated summary statistics under the 2 population sampling strategy. The 3 population sampling strategy serves as a positive control, wherein in an ideal scenario, we would sample all three populations prior to estimating summary statistics.

Under deep divergence models, estimates of Tajima’s *D* (Fig. 5a) did not vary with increased migration among populations. The number of segregating sites (S), Watterson’s *θ*, and nucleotide diversity (*pi*) all had similar patterns across models (Fig. 5b, d, e) such that populations with increased migration from the ghost show greater estimates of all diversity statistics. Estimates of pairwise differentiation between populations ($F_{\mathrm{ST}}$) showed different patterns depending on sampling strategy. A 3 population model reflected higher differentiation among populations where there was no migration between ghost, with differentiation among populations decreasing as migration rates increased (models b–e) (Fig. 5c). A 2 population model did not show any differences in differentiation between sampled populations. $F_{\mathrm{ST}}$ estimates were higher in models of unidirectional gene flow from ghost (models b, c) and lower $F_{\mathrm{ST}}$ in the model with highest bidirectional gene flow (model e) (Fig. 5c). Increasing the number of sampled loci did not change estimated summary statistics, although the differences between high and low migration models were minimized when more loci were sampled (Supplementary Fig. 1).

In comparison, summary statistics did not significantly differ between two- and three-population tests, across all recent divergence models (Supplementary Figs. 46 and 47). This suggests that utilizing measures of population structure and nucleotide diversity are not sufficient to identify the presence of a recently diverged ghost with gene flow between populations. Ranges of both $F_{\mathrm{ST}}$ and *π* for bilateral and unilateral models show estimates within overlapping ranges. We surmise that in recent divergence scenarios, the generational time is not enough to significantly differentiate isolated populations by summary statistics alone. Additionally, permutation tests of the mean $F_{\mathrm{ST}}$ and *π* between the two- and three-population summary statistics of recent divergence models do not yield statistically significant differences (P≥0.4), but permutation tests on deep divergence measurements estimates significant differences (P<0.01) between two- versus three-population tests.

### Effects on the estimation of divergence times

**Fig. 6.**
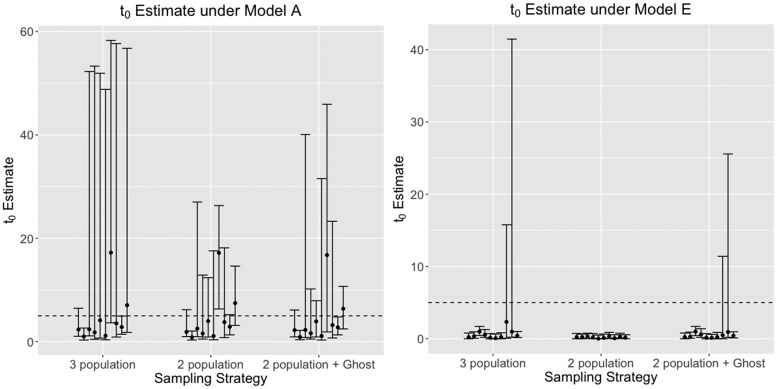
Divergence time ($t_{0}$) estimates between sampled populations, under models a and e, estimated using a 3-population model, 2-population model (without a ghost), and a 2-population model with a ghost outgroup. The dotted line indicates the true (simulated) divergence time ($\frac{t_{0}}{u}=5$, where *u* is the mutation rate per site per generation. Modes, and 95% confidence intervals from 10 replicate runs are shown per model. Compared to the null model a, divergence times are consistently under-estimated with large unidirectional, or bidirectional gene flow from the ghost.

Divergence times ($t_{0}$; true divergence time in years = $\frac{t}{u}$; *u* is the mutation rate per locus per generation) between sampled populations were consistently under-estimated when migration increased from the unsampled ghost population (Fig. 6, Supplementary Figs. 2, 3, and 32), compared to the true values across all our simulations of deep divergence models. Nonetheless, across a majority of our simulations, the simulated divergence time was included in the estimated 95% confidence interval.

Divergence time estimates between the two sampled populations and the ghost population ($t_{1})$ were consistent with the true simulated divergence time ($t_{1}=20$, Supplementary Figs. 4 and 5) with estimates overlapping the 95% confidence intervals. However, the underestimation of $t_{1}$ under a scenario of deep divergence with large bidirectional gene flow (under model e—see Supplementary Figs. 4 and 5) highlights a standing issue under the IM model, as pointed out by Cruickshank and Hahn (2014) and Hey et al. (2015).

### Effects on the estimation of effective population sizes

**Fig. 7.**
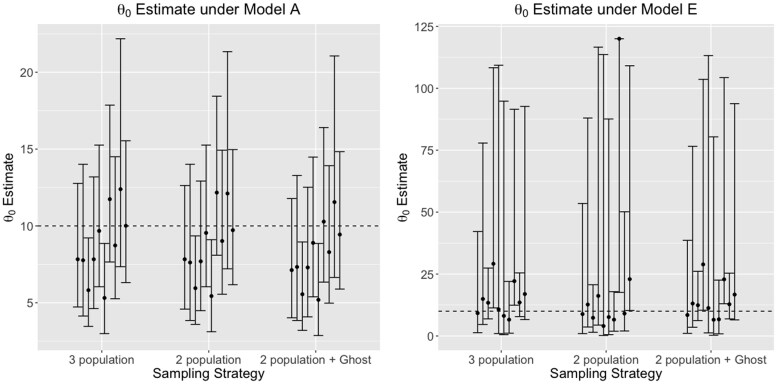
Scaled effective population size ($\theta_{0}=4N_{0}u$) estimate of the first sampled populations under models a and e, estimated using a 3-population model, 2-population model (without a ghost), and a 2-population model with a ghost outgroup. True simulated $\theta_{0}=10$, and is shown with the dotted line. $\theta_{0}$ is consistently over-estimated, as seen by the inflated confidence intervals around the mode, with increased bidirectional gene flow from the ghost (models e), compared to the null model of no gene flow from the ghost (A).

While estimating scaled effective population sizes ($\theta =4N_{e}u$; $N_{e}$ is the effective population size; *u* is the mutation rate per locus per generation) of sampled populations, the inclusion of a ghost population resulted in estimates similar to having sampled all three populations. Nonetheless, greater migration rates (under models c and e) resulted in overestimation of effective population sizes in both sampled populations (Figs. 7, Supplementary Figs. 6–9, 33, and 34).

The level of gene flow from the ghost population greatly affects effective population size estimates of the common ancestor of the two sampled populations (Supplementary Figs. 10, 11, and 35). Estimates from the two population model have smaller confidence intervals when there is no or low migration with the ghost (models a and b), but in models with high migration (c and e), the effective population size was consistently overestimated by ≈2−10 times the “true” value (θ=10).

When estimating the effective population sizes of the common ancestor of the two sampled populations and the ghost population (Supplementary Figs. 14, 15, and 37), the two population model which included the ghost performed as well as the three population model in models of low or no migration (a and b). However in high migration model c, the three population model obtained better estimates, while the model with the inclusion of a ghost mostly underestimated $\theta_{4}$. Under model e (of high bidirectional migration to and from the ghost), the two population model with inclusion of a ghost performed better than the three population model.

### Effects on the estimation of migration rates between populations

**Fig. 8.**
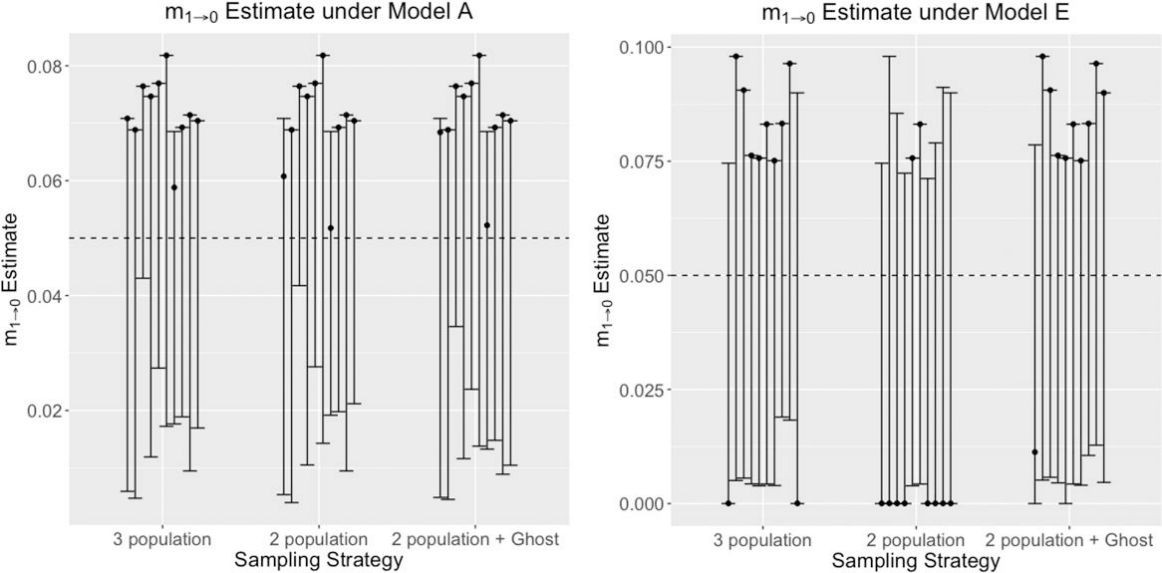
Scaled migration rate ($m_{1\to 0}=4N_{1}u$, where $N_{1}$ is the effective population size of one of the sampled populations, and *u* is the mutation rate per site per generation) estimate between the two sampled populations under models a and e, estimated using a 3-population model, 2-population model (without a ghost), and a 2-population model with a ghost outgroup. True simulated $m_{1\to 0}=0.05$ and is shown with a dotted line. Models with increased gene flow from the ghost consistently lead to over-estimation of $m_{1\to 0}$, as observed by the inflated confidence intervals around the mode.

Migration rates (scaled as m=M/u; *M* is the population migration rate per locus per generation; *u* is the mutation rate per generation per locus) between sampled populations were accurately estimated (Fig. 8, Supplementary Figs. 16–19, 38, and 39). The inclusion of a ghost population did not affect these estimates in two and three population models. Regardless of direction, models with highest true migration rates (C and E) have consistent underestimates of mode of migration rates between sampled populations and the ghost for all models (Supplementary Figs. 20–27, 40–45). Estimates of migration rates in models with lower true migration rates (B and D) are more accurate. Additionally, increasing the number of sampled loci did not improve estimates of migration rates. Nonetheless, estimated confidence intervals do overlap the true migration rates across all simulations.

### Effect of ghost gene flow on African hunter–gatherer evolutionary history

Our estimation of evolutionary history of African hunter–gatherers using two-population IM models, and two-population with a ghost IM models showed results congruent with the four populations + ghost models of Hey et al. (2018).

**Fig. 9.**
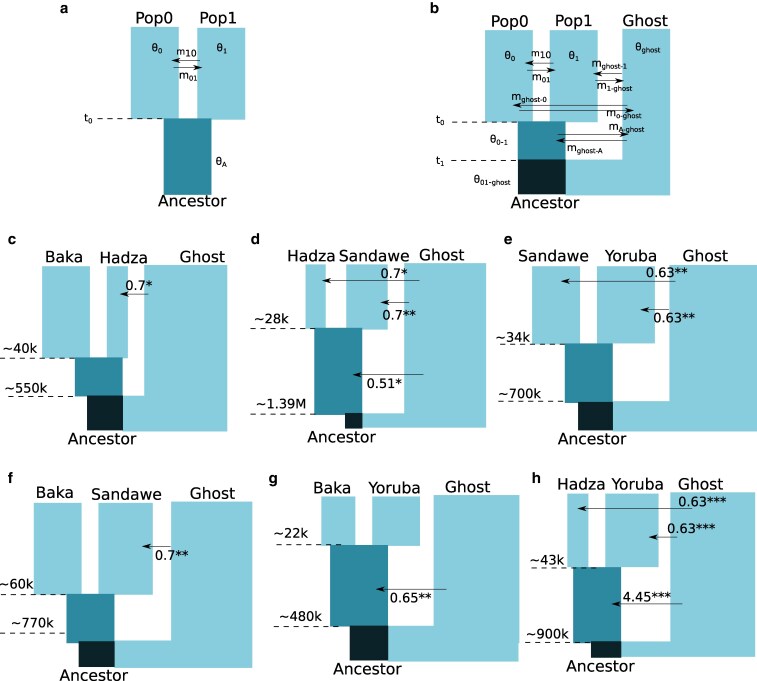
a) Two-population IM model, showing all the estimated parameters—effective population sizes in demographic scales, $\theta_{0},\theta_{1},\theta_{A}$, migration rates ($2N_{e}m$), $m_{10},m_{01}$, and divergence time $t_{0}$ in years before present. b) Two-population IM model with an outgroup unsampled ghost population, with all estimated parameters (effective population sizes, divergence times, and migration rates), c–h) Estimated divergence times in years before present under the 2-population with ghost models between all pairs of populations (Hadza, Sandawe, Baka, and Yoruba). Width of population blocks is proportional to their estimated sizes, while only statistically significant migration rates >0 are shown (* *P* < 0.05, ** *P* < 0.01, *** *P* <0.001). Statistical significance of migration rates were estimated using the LLR test of Nielsen and Wakeley (2001). All parameters were scaled by a human generation time of 29 years.

The Hadza were estimated to have the smallest effective population size across models (≈5,000), while Yoruba were estimated to have the largest effective population size (≈25,000; Fig. 9c–h, Supplementary Tables 2 and 5). All ancestral effective population sizes were estimated to be around 15,000, consistent with previous estimates (Hey et al. 2018). Importantly, no significant migration was detected in all pairwise models by using the LLR test, indicating that there is insufficient information in the genomic data provided to the pairwise model to glean migration estimates (Supplementary Table 1). Divergence time estimates indicated that the earliest split occurred between the Baka and Sandawe populations (≈67,000 ybp), and the most recent split occurred between the Hadza and Sandawe (≈24,000 ybp, Fig. 9c–h, Supplementary Tables 5 and 6).

However, with the addition of an unsampled ghost population to these models, significant migration (assessed using the LLR test of Nielsen and Wakeley 2001) was detected into the extant Hadza, Sandawe, and Yoruba populations (Fig. 9c–h, Supplementary Tables 3 and 4). Common ancestral populations of Hadza–Sandawe, Baka–Yoruba, and Hadza–Yoruba were also estimated to have significant migration from unsampled ghost populations.

Divergence times of common ancestral populations of all tested pairs with the unsampled ghost population was several folds larger than divergence times estimated under the 2-population models (≈483,000 ybp–≈1,39,3800 ybp). Ghost populations were also considerably larger than sampled populations (≈250,000), except between Baka and Sandawe (≈92,000, Fig. 9c–h, Supplementary Table 5).

All two population and two populations with ghost models converged within 48 h, with 100,000 steps of burn-in, which was assessed by similarity of parameter estimates across duplicate runs, as well as using Tracer (Rambaut et al. 2018).

## Discussion

Gene flow from unsampled ghost populations has affected species’ population histories, yet its effect on current evolutionary history models and summary statistics has scarcely been quantified. Here, we develop theoretical expectations for biases in commonly utilized population genetic summary statistics under the Island model. To quantify these biases, we simulate genomic data under the IM model with different levels of gene flow from unsampled ghost populations. Our models reveal that with increased gene flow from unsampled ghosts, differentiation between sampled populations ($F_{\mathrm{ST}}$) is underestimated (Fig. 5c) and genomic diversity (Watterson’s *θ*, nucleotide diversity, *π*) is overestimated. Sampling more genomic loci may improve precision, but it will not eliminate errors or bias. We acknowledge that our simulations have caveats, including sampling a fixed number of individuals from two populations of constant size, primarily owing to the enormity of simulation space. While this limits the generality of our conclusions, we contend that the effects of gene flow from unsampled ghost populations are far from negligible. While we only demonstrate these biases using the IM suite of tools, we contend that similar biases are bound to affect estimates using other tools alike, if ghost populations are not accounted for.

For instance, consider an allopatric speciation scenario (where geographic separation makes two species genomically distinct). If there is more gene flow from an unsampled ghost population, differentiation between the sampled extant populations would be lower than expected under a scenario of no gene flow from the ghost. Investigators may then wrongly conclude that the species are not genetically distinct and that there is gene flow between them when in fact the gene flow occurred in the past between the extant populations and the ghost.

Similarly, not accounting for an unsampled ghost in a model-based estimation of evolutionary history can lead to incorrect conclusions about the true evolutionary history. Our simulations consistently show that when there is an unknown ghost population, current statistical methods will underestimate divergence times, overestimate effect population sizes, and underestimate migration rates between sampled populations.

This affirms previous findings which also showed that not accounting for an unsampled ghost can skew estimates of evolutionary history (Beerli 2004).

Estimates of unidirectional migration rate from one sampled population into the unsampled ghost were always over-estimated when there was no gene flow into the ghost, while under-estimated when there was a larger degree of gene flow into the ghost (e.g. Supplementary Figs. 20 and 21). This could due to the fact that the divergence time between the common ancestor of the sampled populations, and the unsampled ghost population is as yet too small, leading to erroneous conclusions of migration rates between recently diverged populations (Hey et al. 2015).

Increasing the number of sampled genomic loci generally improved the confidence intervals around all estimates. This could be an ideal strategy, especially in this age of next generation sequencing, and the ability to obtain long haplotypic segments at lower costs.

Estimates of effective population sizes were largely robust to the evolutionary model. For instance, in a model with no migration from an unsampled ghost population, the effective population size of the common ancestor of the sampled populations is accurately estimated (Fig. 7).

Our analyses of simulations under recent versus deep divergence scenarios however indicate standing issues of identifiability in estimating migration rates in model-based methods that utilize the IM model. This issue of over-estimation of migration rates between sampled populations under recent divergence or underestimation of migration rates under deep divergence was previously pointed out by Cruickshank and Hahn (2014) and affirmed by Hey et al. (2015). These biases in migration rate estimation therefore persist under scenarios of ghost gene flow.

For instance, in a recent study (Hey et al. 2018) of African hunter–gatherers (Hadza, Sandawe) and agriculturalists (Yoruba, Baka), significant unidirectional migration (ghost into Baka, Yoruba, Sandawe, and the four populations’ common ancestor) was only detected when a ghost population was included in their model. Including the ghost population also produced more accurate estimates of the small effective population sizes in the sampled populations. Both of these patterns also hold in our study.

Our analyses of the demographic history of 20 diploid African Hunter–Gatherer genomes across 355 loci revealed some interesting patterns of population size change, and ancestral introgression, which was also previously detected by summary statistics by Lachance et al. (2012). The Hadza–Sandawe split (Fig. 9d) was determined to be very recent (as recent as 4,500 ybp, 95% c.i. of 4,505–49,560). This concurs with the prior neighbor-joining analyses of Lachance et al. (2012), which indicated the Baka divergence before the Hadza–Sandawe split. Also in agreement with Lachance et al. (2012), all our models determined that the Hadza underwent a recent population bottleneck (with $N_{e}$ = 128–4,300), although estimates are lower than those determined from polymorphism levels ($N_{e}$ = 9,200–20,900 Lachance et al. 2012). Significant ancestral introgression with an unsampled ghost population was also detected, but was largely unidirectional (from the ghost into extant populations), suggesting that introgression events were more ancient than divergence from recent common ancestors of extant populations. Divergence times with this ancestral ghost population were as high as 1.7 million years ago, which was also previously detected by Lachance et al. (2012) using TMRCA estimates on introgressed regions. Importantly, if a researcher were to only utilize 2-population IM models to infer the evolutionary history of the Hadza, Sandawe, Yoruba, and Baka, their inference would be at odds with the estimated population structure and admixture proportions of Lachance et al. (2012). The addition of an unsampled ghost however to the 2-population IM model in IMa3 correctly infers their evolutionary history, congruent with the findings of Hey et al. (2018).

Our study reiterates the key points made by Beerli (2004), in that while it is impossible to always sample all species or large number of informative genomic loci, it is imperative to account for the presence of unsampled ghost populations while estimating both summary statistics and the evolutionary history of sampled species.

Detecting the presence of a ghost population in genomic data should ideally be a multipronged approach: (i) computing summary statistics from population genetic data, and reconciling observed summary statistics with the species’ known biological history, (ii) estimating the population genetic structure of sampled populations via a model-based approach like STRUCTURE or ADMIXTURE (Pritchard et al. 2000; Alexander et al. 2009), (iii) if available, using genome-wide estimates of summary statistics like differentiation (e.g. $F_{\mathrm{st}}$) and divergence ($D_{xy}$) between populations to identify regions of low differentiation or divergence, and hence putatively introgressed from an recently divergend unsampled ghost, or regions of high differentiation or divergence to identify regions of introgression from a deeply diverged unsampled ghost, (iv) computing TMRCA (Time to Most Recent Common Ancestry) distribution across the genome, sensu (Lachance et al. 2012), to visualize differences in coalescent time distributions of putatively introgressed (from archaic/ghost populations) regions, versus nonintrogressed regions, and finally, (v) testing a variety of evolutionary models, both including and excluding the presence of an unsampled ghost under a model-based framework, like ABC (Approximate Bayesian Computation Beaumont et al. 2002), or IM (Hey and Nielsen 2004) to find the best fitting model that explains observed population genomic data.

Alternately, in the absence of genome-size data, for e.g. performing analyses with a few microsatellite or nuclear loci), our recommendation would still be to begin with steps (i) and (ii) above, and then, estimate evolutionary history using a variety of models with and without ghost populations using the LLR tests implemented in IMa2p and IMa3. Additionally, both programs allow for conveniently testing of significant nonzero bidirectional migration estimates from an outgroup ghost using the LLR test of Nielsen and Wakeley (2001).

## Supplementary Material

jkaf180_Supplementary_Data

## Data Availability

All simulation scripts, IMa3 datasets are available on the project’s GitHub page at https://github.com/ClassicalKooz/GhostPopulationSimulation. Briefly, this GitHub page is structured with separate folders for “Deep” and “Recent” divergence simulations, plots (presented in this manuscript under “Graphs”), as well as scripts for simulations and computing summary statistics (“Scripts”). The empirical datasets analyzed were collated from the studies of Lachance et al. (2012) and Fan et al. (2019) and are made available at https://github.com/arunsethuraman/huntergatherer_popgen. Supplemental material available at G3 online.
